# Relationship of Sanitation Parameters with Microbial Diversity and Load in Raw Meat from the Outlets of the Metropolitan City Biratnagar, Nepal

**DOI:** 10.1155/2019/3547072

**Published:** 2019-03-03

**Authors:** Sanjay Mahato

**Affiliations:** ^1^AASRA Research and Education Academic Counsel, Janpriya Tole, Biratnagar-6, Nepal; ^2^Department of Microbiology, Mahendra Morang Adarsha Multiple Campus, Tribhuvan University, Biratnagar, Nepal

## Abstract

The main aim of this study is to assess the microbial load of raw meat from outlets of Biratnagar and its relationship with several sanitation parameters. Samples were taken from meat outlets, and required microbiological procedures were followed as per guidelines. Approximately 63.6% of microbes were present in meat with poor sanitation while 36.4% were present in meat with good sanitation. Fungal contamination in poorly kept mutton was one and half times greater than chicken/mutton of good sanitation. Fungi such as *Penicillium* (21.3%), *Mucor* (16.3%), *Aspergillus* (15%), and *Trichosporon* (13.8%) were most predominant. 73.8% of meat samples contained *Staphylococcus* spp., 61.3% contained *E. coli,* 48.8% of *Pseudomonas* spp., and 37.5% samples contained *Salmonella* spp. Outlets selling both types of meat showed no significant difference in microbial types. Mean of TVC of meat is 8.2 log CFU/g. Mean TVC of mutton (7.6 log CFU/g) is lower than mean TVC of chicken/meat (8.5 log CFU/g) and differed significantly. Tiled outlets showed comparatively lower bacterial contamination than cemented outlets which was statistically significant (*t* = −3.16, *p*=0.002). With the difference among microbial type and few sanitation parameters being statistically significant, it can be suggested that outlets should be tiled (*p*=0.002), showcased (*p*=0.001), and the meat-handling employee must wear washed apron (*p*=0.013). Proper cleaning of water supply and use area (*p* ≤ 0.001) and drainage (*p*=0.048) maintain a good meat sanitation (*p* ≤ 0.001) which reduces microbial contamination significantly. To diminish microbiological load on meat sold in the Biratnagar city, standard operating methods should be practiced.

## 1. Introduction

According to Heinz and Hautzinger [1], the Canadian annual per capita consumption of meat increased from 10 kg in the 1960s to 26 kg in 2000 and will reach 37 kg by the year 2030. As per Ministry of Agriculture and Development of Nepal, it has annual per capita consumption of 11.1 kg. A significant portion of meat and meat products are spoiled every year [2] at the consumer, retailer, and foodservice levels which have a substantial economic and environmental impact. A significant portion of this loss is due to microbial spoilage [3]. Livestock are often found being slaughtered for their meat in dirty, foul-smelling areas. The meat distribution is found to be violating the Animal Slaughterhouse and Meat Inspection Act, 1999, Kathmandu [4].

Inadequacy of different technical operations at any stage of slaughtering such as stunning, bleeding, skinning, evisceration, and carcass splitting will result in a rigorous negative impact on the product [5]. Hygiene, storage temperature, the acidity of the meat, and the structure of the muscular tissue affect the rate of meat spoilage. Liver spoils faster than the firm muscular tissue of beef [6]. Rigor mortis affected by stress conditions during slaughtering process affects the meat quality [7, 8]. Fat, protein, minerals, carbohydrate, and water present in meat [1] degrade because of digestive enzymes, microbial spoilage, and lipid oxidation [6]. Meat has a pH of 6.2–6.8, and the meat having lower value of pH is responsible for the breakdown of proteins, providing a favorable medium for the growth of bacteria [8, 9].

Meat and meat products provide excellent growth media for bacteria, yeasts, and molds, some of which are pathogens [10]. The skin and the intestinal tract of the animal are the chief sources of these microorganisms. The composition of microflora in meat depends on various factors such as preslaughter husbandry practices (free range vs intensive rearing), age of the animal at the time of slaughtering, handling during slaughtering, evisceration and processing, temperature controls during slaughtering, processing and distribution, preservation methods, type of packaging, and handling and storage by consumer [3]. Mold species include *Cladosporium*, *Sporotrichum*, *Geotrichum*, *Penicillium, Alternaria,* and *Monilia* and *Mucor* while yeasts species include *Candida* spp., *Cryptococcus* spp., and *Rhodotorula* spp. [11]. Bacterial species include *Pseudomonas*, *Acinetobacter*, *Morexella, Alkaligenes, Micrococcus, Streptococcus, Sarcina, Leuconostoc*, *Lactobacillus, Proteus, Flavobacterium, Bacillus*, *Clostridium, Escherichia*, *Campylobacter, Salmonella, Streptomyces*, *Shigella, Staphylococcus, Yersinia, Listeria, Acrobacter, Mycobacterium,* and *Bacillus* [12–14]. Many of these bacteria can grow in chilling temperatures.

*Pseudomonas* spp. are Gram-negative, nonfermentative rods, aerobic, and motile with polar flagella [10, 15] which are present everywhere including drinking water, domestic and wild animals, human beings, plants and also in a variety of foods. *E. coli* has been isolated worldwide from poultry meat which might be due to fecal contamination [16, 17]. Enteroinvasive, enteropathogenic, and enterotoxigenic types of *E. coli* can be a foremost cause of foodborne diarrhoea [18]. Transmission of *Vibrio cholerae* to humans occurs through ingesting contaminated water or food especially poultry products [19]. Contamination of meat by *Staphylococcus* species may occur during the phase of manufacturing and handling of final products [20]. *Salmonella* is the most incriminated pathogenic microorganisms of bacterial food poisoning especially present in poultry meat, with infection being through the handling of raw poultry carcasses and products, together with the consumption of undercooked poultry meat [21]. *Shigella* species have highly evolved invasive systems that cause bacillary dysentery or shigellosis [22].

The main aim of this study is to assess the microbial load of raw meat from outlets of Biratnagar and to understand its possible role in spoilage and foodborne illnesses. The study also statistically analyzes the relationship among sanitation parameters, microbial load, microbial diversity, and microbial type.

## 2. Materials and Methods

### 2.1. Study Area

This cross-sectional study was carried out in the Biratnagar city of Morang district, lying in the Terai region of Nepal from 2017 January to 2018 July. Random sampling was done to collect nonrepeated single meat samples from different meat outlets located in different places of Biratnagar. The experiments were carried out at Microbiology Laboratory of Mahendra Morang Adarsh Multiple Campus, Biratnagar. Each outlet in this study also slaughtered animals to sell meat in its outlet. In the slaughtering house, bleeding and skinning were done on the floor, and both procedures were performed by the same workers with the same knives. In the slaughtering house, hot water (80°C–90°C) was used during skinning process. Knives were not washed while cutting the different types of meat. Knives were washed only after the work used to stop for a longer period (>1 hr).

### 2.2. Sampling and Sample Processing

Sanitation parameters such as tiled or cemented; washing of slaughterhouse, apron, and chopping boards; uses of hand sanitizer; hygienic condition of slaughterhouse; water supply area and drainage; showcased meat; and cleanliness of slaughter personnel were selected, and on these bases, meat samples were categorized as good sanitation and poor sanitation types. Of 80 collected samples, 40 samples (20 mutton and 20 chicken) were grouped into good sanitation type and 40 samples (20 mutton and 20 chicken) were grouped into poor sanitation type.

A total of 80 meat samples collected from 40 outlets (40 chicken and 40 mutton) were processed. All the 40 outlets selected randomly were categorized as selling both kind of meat. Forty fresh samples of raw chicken meat (20 g; wing's part) and forty samples of raw mutton (20 g; thigh meat) were collected aseptically in a sterile capped plastic container from different meat shops and were transported to the laboratory in a icebox for microbial analysis within 1 hour of sampling. Sterile containers were used for each meat sample.

Twenty grams of the collected meat sample were measured and were aseptically cut into thin smaller pieces using a sterile knife, and the meat pieces were kept in a sterile conical flask containing 80 ml buffered peptone water (HiMedia, M028) and were incubated for 30 min and shaken vigorously on an interval of 5 min. Thus, a 10^−1^ dilution was prepared. A further dilution till 10^−6^ was prepared.

### 2.3. Isolation and Enumeration of Microbes

The rinsate was inoculated on total plate count agar by the spread plate method to enumerate TVC. Plates were incubated at 37°C for 24 hrs. For isolation of *E. coli* and *Salmonella* spp., Eosin methylene blue (EMB) agar (HiMedia, Mumbai, India) plate and Xylose Lysine Deoxycholate Agar (XLDA) (HiMedia, Mumbai, India) plate were spread with 0.1 ml inoculum from several dilutions, respectively, and incubated at 37°C for 24 hrs and consequently subcultured onto nutrient agar (HiMedia, Mumbai, India) plate to get pure culture for further identification. Similarly, Cetrimide agar plate (HiMedia, Mumbai, India), thiosulfate-citrate-bile salts-sucrose (TCBS) agar plate (HiMedia, Mumbai, India), and Mannitol salt agar (MSA) plate (HiMedia, Mumbai, India) were used for cultivation of *Pseudomonas* spp., *Vibrio* spp., and *Staphylococcus* spp., respectively. For fungi isolation, inoculum was spread on Sabouraud Dextrose Agar (SDA) (HiMedia, Mumbai, India) plate and incubated at 25°C–27°C for 48 hrs.

Characterization and identification of the colony isolates were achieved by initial morphological examination of the colonies in the plate (macroscopically) for colonial appearance, size, elevation, form, edge, consistency, color, odor, opacity, and pigmentation. Gram staining, capsule staining, flagellar staining, and spore staining were essentially performed on isolates from the colonies as a preliminary identification of bacteria. Fungal molds and yeasts were identified by performing lactophenol cotton blue staining [23]. The bacterial isolates were identified by cultural, physiological, morphological, and biochemical tests as stated by Bergey's Manual of Determinative Bacteriology [24]. Biochemical characterization of the bacteria was done by performing specific tests such as catalase, oxidase, TSI, indole, methyl red, Voges–Proskauer and citrate tests, carbohydrate fermentation tests, coagulase, O/F tests, and urease test [25].

### 2.4. Data Analysis and Statistical Tools

The data were statistically analyzed using the Statistical Package the for Social Sciences (SPSS v21) software package. Data frequencies and cross tabulations were used to summarize descriptive statistics. Tables were used for data presentation. The chi-square test was performed on the data at a level of significance of 5%. The null hypothesis was that factors of sanitation parameter did not influence the types of microbes found in meat samples. For this purpose, the Pearson chi-square value or Fisher's exact test was adopted (whichever was suitable). The independent *T*-test and Pearson correlation test were performed on the obtained data.

## 3. Results

Out of 13 target microorganisms, a total of 132 isolates (100 bacteria + 32 fungi) were identified from 40 chicken samples while 129 (92 bacteria + 37 fungi) isolates were counted in 40 mutton samples (Table 1). Only one isolate was selected from one culture plate which compounded to a total of 261 isolates from 80 meat samples. Six (5 mutton + 1 chicken) samples showed growth with single microbe while remaining samples showed growth with multimicrobial growth. Mean of number of isolate types found in meat sample was 3.26 (chicken 3.3; mutton 3.23).

**Figure 1:**
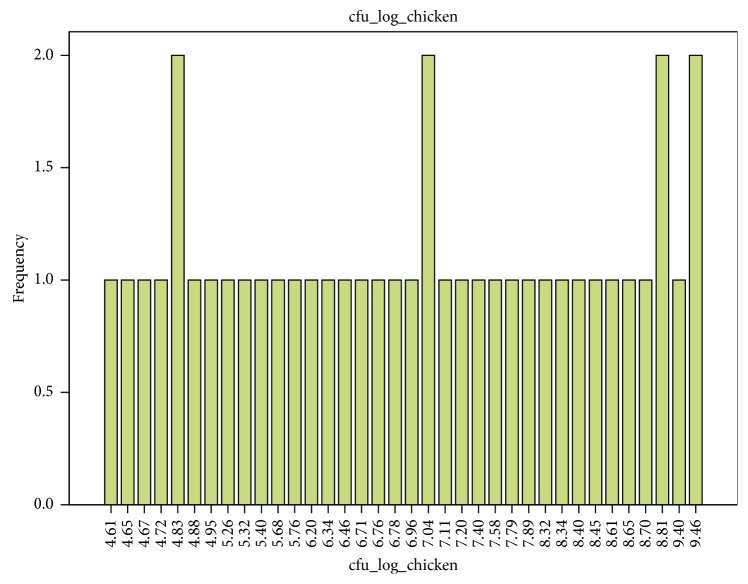
Total viable count in chicken.

**Figure 2:**
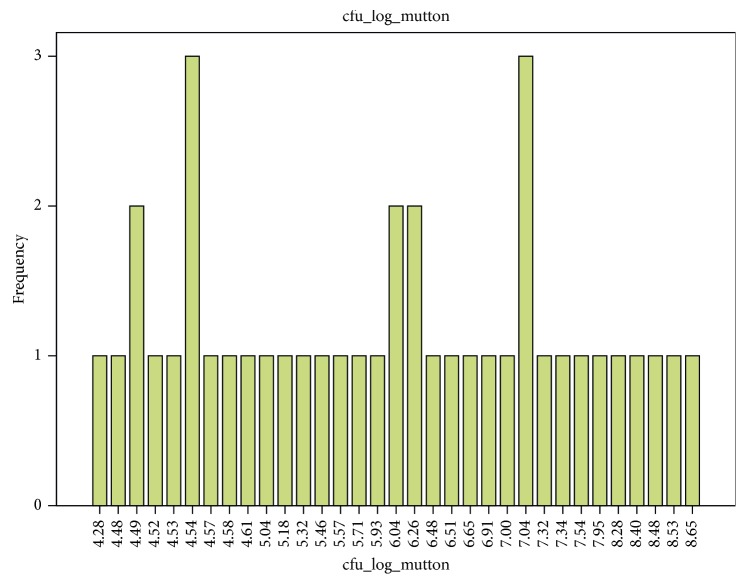
Total viable count in mutton.

The TVC of meat was 8.2 log CFU/g. The TVC of chicken meat ranged from 4.6 log CFU/g to 9.5 log CFU/g for all chicken meat examined (Table 2). The TVC of mutton ranged from 4.3 log CFU/g to 8.7 log CFU/g. Mean TVC of mutton 7.6 log CFU/g from outlets were lower than mean TVC of chicken meat 8.5 log CFU/g (Figures 1 and 2). There were no significant differences (*p* ≤ 0.05) between the TVC of mutton and chicken meat.

Based on sanitation parameters (tiled or cemented; washing of slaughterhouse, apron, and chopping boards; uses of hand sanitizer; hygienic condition of slaughter house; water supply area and drainage; showcased meat; and cleanliness of slaughter personnel), meat samples were categorized as good sanitation and poor sanitation types. Out of 80 collected samples (40 mutton and 40 chicken), 40 samples (20 mutton and 20 chicken) were grouped into good sanitation type and 40 samples (20 mutton and 20 chicken) were grouped into poor sanitation type. Four (10%) of the outlets had improper washing while 12 (30%) of the outlet personnel wore unwashed apron (Table 3). Eight (20%) of the chopping box was in unwashed condition while taking the sample. Interestingly, none of the outlets washed the chopping board when chicken and mutton were alternately chopped. Neither of the meat-handling personnel used hand sanitizer. Nine (45%) of the outlet meat were kept in open and were not covered. Seventeen (42.5%) of the slaughter area had improperly cleaned water supply area, while 40% of outlets had no proper drainage facility.

Out of 261 isolates, 95 (36.4%) and 166 (63.6%) microbial isolates were obtained from good sanitation and poor sanitation type meat samples, respectively (Table 3). Among 95 isolates from good sanitation, 37 bacterial and 15 fungal isolates were of chicken while 29 bacterial and 14 fungal isolates were from mutton. Among 166 isolates from poor sanitation, 63 bacterial and 17 fungal isolates were from chicken while 63 bacterial and 23 fungal isolates were from mutton.

**Figure 3:**
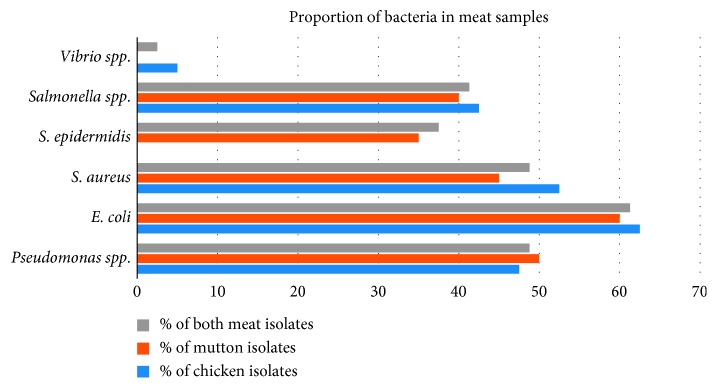
Proportion of bacteria in meat samples.

**Figure 4:**
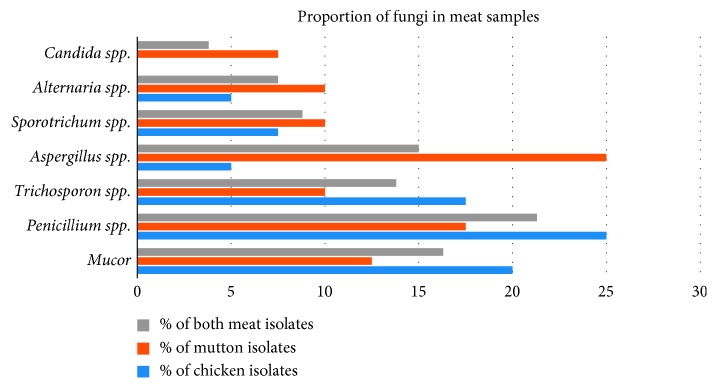
Proportion of fungi in meat samples.

Most prevalent bacterial contamination was of *Staphylococcus* spp. followed by *E. coli*, *Pseudomonas* spp., and *Salmonella* spp. Among 80 samples, 73.8% of meat samples contained *Staphylococcus* spp., 61.3% of samples contained *E. coli,* 48.8% of samples contained *Pseudomonas* spp., and 37.5% samples contained *Salmonella* spp. (Table 1; Figure 3). *Vibrio* spp. were present in only 2.5% samples. Among 69 fungal isolates, the number of *Penicillium* spp., *Mucor*, *Aspergillus* spp., *Trichosporon* spp., *Sporotrichum* spp., *Alternaria* spp., and *Candida* spp. were 17 (21.3%), 13 (16.3%), 12 (15%), 11 (13.8%), 7 (8.8%), 6 (7.5%), and 3 (3.8%), respectively (Table 1; Figure 4).

From the analysis of presence of microbial isolates in meat samples against sanitation parameters, it was evident that microbial load was much higher in cemented outlets, improperly washed slaughter area, unwashed chopping box, personnel wearing unwashed apron, uncovered meat, improperly cleaned water supply area, improper drainage facility, and poorly sanitated meat. *E. coli* was the foremost bacteria found in most of the sanitation parameters such as improperly washed slaughter house (87.5%) when compared to properly washed (58.3%); unwashed apron (70.8%) vs washed apron (57.1%); unwashed chopping box (75%) vs washed (57.8%); showcased meat (72.2%) vs uncovered (52.3%); improper drainage (71.9%) vs proper (54.2%); cemented outlets (77.5%), improperly cleaned water supply area (85.3%), and poor sanitation meat (85%) (Tables 4–10). *Pseudomonas* spp. contamination sharply decreased with proper sanitation measures. Staphylococcal contamination inconveniently reduced/increased in proper sanitation measures taken (Tables 4–6). *Salmonella* spp. was present next to *E. coli* and *Pseudomonas* spp. in improperly washed slaughter house (50%), unwashed apron (58.3%), unwashed chopping box (62.5%), and improperly maintained drainage (62.6%). This study showed that proper washing of aforementioned parameters highly reduced its presence in meat. *Vibrio* spp. was barely present in samples which were specific to cemented outlets, improperly cleaned slaughter, and water supply area (Tables 4–6).

*Mucor* and *Penicillium* were among the highly found fungal contaminants in meat samples. Improper cleaning and maintenance of water supply area (32.4%) and drainage (18.8%) and poorly kept meat (27.5%) showed high amount of *Mucor* which decreased sharply with proper sanitation measures (Tables 4–6). Surprisingly in few cases, *Mucor* contamination was found to be higher after proper measures such as slaughter house wash (16.7%), apron wash (17.9%), and chopping box wash (20.3%). In the same manner, *Penicillium* increase was observed in showcased meat (27.3%), properly cleaned water supply (21.7%), proper drainage (22.9%), and good sanitation meat (25%). *Trichosporon* spp. was also found in greater number next to *Mucor* and *Penicillium.*

### 3.1. Statistical Analysis

The chi-square test indicated that there is a strong evidence that *Pseudomonas* contamination was significantly dependent on types of outlet (*χ*^2^ value = 8.455, df = 1, *p*=0.004), showcase use (*χ*^2^ = 11.22, df = 1, *p* ≤ 0.001), water supply area (*χ*^2^ = 14.532, df = 1, *p* ≤ 0.001), and meat sanitation type (*χ*^2^ = 18.061, df = 1, *p* ≤ 0.001) (Table 9). *E. coli* contamination was significantly dependent on types of outlet (*χ*^2^ value = 8.901, df = 1, *p* ≤ 0.001), water supply area (*χ*^2^ = 14.403, df = 1, *p* ≤ 0.001), and meat sanitation type (*χ*^2^ = 19.013, df = 1, *p* ≤ 0.001). *Staphylococcus aureus* contamination was significantly dependent on showcase use (*χ*^2^ = 8.41, df = 1, *p* ≤ 0.004) and meat sanitation type (*χ*^2^ = 6.054, df = 1, *p*=0.014). *Staphylococcusepidermidis* was significantly dependent on water supply area (*χ*^2^ = 7.216, df = 1, *p*=0.007) and meat sanitation type (*χ*^2^ = 18.061, df = 1, *p* ≤ 0.001). *Salmonella* contamination was significantly dependent on types of outlet (*χ*^2^ = 8.717, df = 1, *p*=0.003), washing of apron (*χ*^2^ = 4.129, df = 1, *p*=0.042), water supply area (*χ*^2^ = 10.269, df = 1, *p*=0.001), drainage (*χ*^2^ = 13.075, df = 1, *p* ≤ 0.001), and meat sanitation type (*χ*^2^ = 8.717, df = 1, *p*=0.003). *Vibrio* spp. and several fungi in study such as *Trichosporon*, *Aspergillus*, *Sporotrichum*, *Alternaria*, and *Candida* were independent of any of the sanitation parameters. *Mucor* contamination was significantly dependent on types of outlet (*χ*^2^ = 4.501, df = 1, *p*=0.034), showcase use (*χ*^2^ = 9.843, df = 1, *p*=0.002), water supply area (*χ*^2^ = 11.266, df = 1, *p*=0.001), and meat sanitation type (*χ*^2^ = 7.44, df = 1, *p*=0.006). *Penicillium* contamination was significantly dependent on types of outlet (*χ*^2^ value = 9.038, df = 1, *p*=0.33). Tables 7 and 8 present the chi-square test of different microbial isolates of chicken and mutton meat against several sanitation parameters, respectively.

Pearson correlation between TVC of meat and the number of isolate types of meat was highly significant (*r* = 0.719, *p* ≤ 0.001). Pearson correlation between the number of isolate types of chicken and mutton was highly significant (*r* = 0.745, *p* ≤ 0.001). Pearson correlation between the TVC of chicken and mutton was highly significant (*r* = 0.816, *p* ≤ 0.001). The relationship of the number of isolates of chicken with TVC of chicken was significant (*r* = 0.701, *p* ≤ 0.001) like the number of isolates of mutton with TVC of mutton (*r* = 0.797, *p* ≤ 0.001).

The independent *T*-test illustrated that the mean of isolates from tiled outlet is significantly different from the cemented outlet (*t* = −3.160, d.f. = 78, *p* ≤ 0.002). Similarly, washed apron, covered meat, properly cleaned water supply area, proper drainage, and good meat sanitation showed significant difference of microbe type and number against improperly washed apron, uncovered meat, improperly cleaned water supply area, improper drainage, and bad meat sanitation, respectively (Table 10). The independent *T*-test proved that the mean TVC from tiled outlet is significantly different from the cemented outlet (*t* = −2.736, d.f. = 78, *p*=0.008). Similarly, covered meat, properly cleaned water supply area, and good meat sanitation showed significant difference of TVC against uncovered meat, improperly cleaned water supply area, and bad meat sanitation, respectively (Table 11). Mean TVC of chicken is significantly different from mean TVC of mutton (*t* = 2.43, d.f. = 78, *p*=0.017).

## 4. Discussion

Presence of nearly equal number of isolates, i.e., 132 isolates (100 bacteria and 32 fungi) from 40 chicken samples and 129 (92 bacteria and 37 fungi) isolates from 40 mutton samples and from two different types of meat, namely, mutton and chicken from the same outlet clearly indicated the cross contamination of microbes [26]. This can be because of using same chopping board, chopping knife, and unwashed hand for both types of meat [26]. Nearly, 36.4% microbial isolates from good sanitation and 63.6% isolates from poor sanitation type meat highlight the importance of proper hygiene and sanitation parameters for reducing microbial contamination.

TVC is a broadly accepted measure of the general degree of microbial contamination and hygienic conditions of processing plants or outlets [27]. The TVC of meat was 8.2 log CFU/g. There were no significant differences (*t* (78) = 2.43, *p* ≤ 0.05) between the TVC of mutton and chicken meat. TVC of mutton and chicken (7.6 log CFU/g and 8.5 log CFU/g) was higher (6.62 log CFU/g and 7.22 log CFU/g) than the findings in Lahore, Pakistan [28], and by Selvan et al. [29] for mutton (5.35 log CFU/g) and chicken (4.52 log CFU/g).

*E. coli* in mutton and chicken was higher than Lahore, Pakistan [28], while much lower than Kolkata, India [30]. *E. coli* was the foremost bacteria found in most of the sanitation parameters such as improperly washed slaughter house, unwashed apron, unwashed chopping box, improper drainage, cemented outlets, improperly cleaned water supply area, and poor sanitation meat. *E. coli* contamination was significantly dependent on types of outlet (*χ*^2^ = 8.901, *p* ≤ 0.003), water supply area (*χ*^2^ = 14.403, *p* ≤ 0.001), and meat sanitation type (*χ*^2^ = 19.013, *p*=0.001). The high level of *E. coli* contamination could be due to poor handling by retailers, exposure to direct air and flies, transport vehicle used, and ineffective washing activities. A potential cause of foodborne diseases, i.e., *E. coli*, shows higher levels of contamination in meat which could be attributed to the fact that meat offers a rich nutrient media for microbial growth [31]. Not only as an indicator organism of sanitary quality, *E. coli* is also used as an index organism of pathogens. *E. coli* originates primarily from the intestines of birds and animals, and to a lesser extent, from workers or environment of the processing plant [32]. The growth of these organisms can be controlled by minimizing contamination of slaughtered meat from intestinal contents, following good sanitary practices, and considering time-temperature control of product at retail.

*Staphylococcus aureus* in chicken and mutton was 52.5% and 45% which was much higher than Kolkata, India (22% and 18%) [30], and comparatively lower than Lahore [28]. Not only *Pseudomonas,* but also *Staphylococcus epidermidis* in chicken and mutton (40% and 35%) also was higher than the study (17% and 23%) of Sharma and Chattopadhyay in 2015 [30].

*Staphylococcus aureus* contamination was significantly dependent on showcase use ((*χ*^2^ = 8.41, *p*=0.004) and meat sanitation type ((*χ*^2^ = 6.054, *p*=0.014) while *Staphylococcus epidermidis* was significantly dependent on water supply area ((*χ*^2^ = 7.216, *p*=0.007). *Staphylococcus aureus* is a normal resident of the chickens, located on the skin and feathers and in the respiratory and intestinal tracts (Bennett, 1996). Staphylococcal contamination inconveniently reduced/increased in proper sanitation measures taken. During slaughtering, *Staphylococcus aureus* contamination could gain entry from high poultry concentration, slaughtering and processing equipment, and business devices, through sneezing, coughing, breathing, or talking [32], and from the processes of scalding and evisceration, due to cross contamination, are responsible for increased *Staphylococcus aureus* contamination [20]. The load of *Staphylococcus aureus* in poultry and meat reflects the level of hygiene of the handler [27].

*Pseudomonas* spp. contamination sharply decreased with proper sanitation measures. *Pseudomonas* contamination was significantly dependent on types of outlet (*χ*^2^ = 8.455, *p* ≤ 0.004), showcase use (*χ*^2^ = 11.22, *p* ≤ 0.001), water supply area (*χ*^2^ = 14.532, *p* ≤ 0.001), and meat sanitation type (*χ*^2^ = 8.455, *p*=0.001). *Pseudomonas* spp. are recognized as major food spoilers [7], and they are psychrotrophic bacteria that easily develop in foods stored aerobically like meat, fish, milk, and dairy products [33]. *Pseudomonas* increases in levels from the environment to meat because the meat matrix provides more favorable conditions to grow and become the dominant population [34].

The prevalence of *Salmonella* spp. was 41.3% which is similar to the studies carried out in China and Colombia [35, 36]. On the contrary, the higher prevalence rate of *Salmonella* spp. was found in Southern Thailand (67.5%) [37]. Contamination of chicken meat with *Salmonella* spp. may occur during slaughtering process or evisceration [38]. It can be a cause of foodborne salmonellosis and meat spoilage [39, 40]. *Salmonella* spp. was present in improperly washed slaughter house, unwashed apron, unwashed chopping box, and improperly maintained drainage. *Salmonella* contamination was significantly dependent on types of outlet (*χ*^2^ = 8.717, *p* ≤ 0.003), washing of apron (*χ*^2^ = 4.129, *p* ≤ 0.042), water supply area (*χ*^2^ = 10.269, *p* ≤ 0.001), drainage (*χ*^2^ = 13.075, *p*=0.001), and meat sanitation type (*χ*^2^ = 8.717, *p* ≤ 0.003). *Salmonella* spp. might have contaminated the meats because of poor handling by meat sellers, contamination from the water used by the retailer in washing the produce, its exposure to direct air, and also from the tables of the retailers from which produce is displayed [41]. In addition, the size and structure of the market could also contribute to the increased incidence of *Salmonella* contamination. Highly populated area with compactness of sellers and consumers can upsurge microbial contamination by the skin, mouth, or nose of the handlers and consumers which might be introduced directly into the meat [42].

*Vibrio* spp. was absent in the mutton sample which was similar to the work done in Libya [43]. For chicken samples, the finding was very low (5%) compared to 55.5% [43]. *Vibrio* spp. was barely present in samples which were specific to cemented outlets, improperly cleaned slaughter, and water supply area.

The predominant mold pathogen isolated from meat was *Penicillium* spp. (21.3%), *Mucor* (16.3%), *Aspergillus* spp. (15%), *Sporotrichum* spp. (8.8%), and *Alternaria* spp. (7.5%) which were greater than the findings in Chennai, India [44]. The predominant yeast pathogen isolated was *Trichosporon* spp. (13.8%) and *Candida* spp. (3.8%) which were in agreement with Thanigaivel and Anandhan [44].

Molds such as *Mucor* and *Penicillium* and yeasts such as *Trichosporon* were highly found fungal contaminants in meat samples. Improper cleaning and maintenance of water supply area and drainage and poorly kept meat showed high amount of *Mucor* which decreased sharply with proper sanitation measures. *Mucor* contamination was significantly dependent on types of outlet (*χ*^2^ = 4.501, *p* ≤ 0.034), showcase use (*χ*^2^ = 9.843, *p* ≤ 0.002), water supply area (*χ*^2^ = 11.266, *p* ≤ 0.001), and meat sanitation type (*χ*^2^ = 7.44, *p* ≤ 0.006). Surprisingly, little *Mucor* contamination was found to be higher after proper measures such as slaughter house wash, apron wash, and chopping box wash. This could be better explained by the findings of Barnes et al. [45]. During slaughtering the feathers, feed and bodies of the birds, and outsides of cages have also been found to be contaminated with yeasts [46]. The air and soil of poultry breeding and rearing houses, old litter and litter containing water, wet feed, and bird droppings have been found to contain yeasts [47]. From the results, it would appear that yeasts are considerably represented in the total microbial ecology of poultry carcasses, although yeasts are rarely the direct cause for spoilage [48].

From the analysis of microbial isolates found in meat samples against sanitation parameters, it was evident that microbial load was higher in cemented outlets, improperly washed slaughter area and chopping box, meat handlers wearing unwashed apron, open meat, improperly cleaned water supply area, improper drainage facility, and poorly sanitated meat. Undoubtedly, the meat outlets in the Biratnagar city may carry high initial microbial contamination from the point of slaughtering process to the point of offering to consumers. Biomagnifications occur at all levels of handling, poor transport, and retailing conditions [49]. The mean of isolates from the tiled outlet is significantly different from the cemented outlet (*t* (78) = −3.16, *p*=0.002) which indicates that tiling of outlets is better than cemented outlets to reduce microbial load.

## 5. Conclusion

It is concluded that microbial load of raw meat from outlets in Biratnagar is high which insinuates its possible role in spoilage and foodborne illnesses. The exposure of meat products to unhygienic practices from the point of production to retail level increases the level of microbial contamination in the produce. To diminish microbiological load on meat carcasses sold in the Biratnagar Metropolitan City, standard operating methods should be practiced. Such methods include more stringent inspection, regular supervision and/or monitoring of hygiene practices, regular interval screening of butchers, meat sellers, and all people involved in handling of meat. In addition, properly tiled outlets, well-maintained meat chopping box, selling tables covered with nets, thoroughly cleaned and regularly sterilized knives, aprons, and all the equipment that meats encounter should be used. Further research should be done to assess the meat safety and hygiene knowledge levels of meat handlers, the bacterial load on meat at the abattoir and butchery levels.

## Figures and Tables

**Table 1:** Type of microbes isolated from chicken, mutton, and meat.

S. No.	Isolates	Chicken isolates		Mutton isolates		Total meat isolates	
		Number (*n*)	Percentage (%)	Number (*n*)	Percentage (%)	Number (*n*)	Percentage (%)
1	*Pseudomonas* spp.	19	47.5	20	50	39	48.8
2	*E. coli*	25	62.5	24	60	49	61.3
3	*S. aureus*	21	52.5	18	45	39	48.8
4	*S. epidermidis*	16	40	14	35	30	37.5
5	*Salmonella* spp.	17	42.5	16	40	33	41.3
6	*Vibrio* spp.	2	5	0	0	2	2.5
7	*Mucor*	8	20	5	12.5	13	16.3
8	*Penicillium* spp.	10	25	7	17.5	17	21.3
9	*Alternaria* spp.	2	5	4	10	6	7.5
10	*Aspergillus* spp.	2	5	10	25	12	15
11	*Sporotrichum* spp.	3	7.5	4	10	7	8.8
12	*Trichosporon* spp.	7	17.5	4	10	11	13.8
13	*Candida* spp.	0	0	3	7.5	3	3.8
Total		132	—	129	—	261	—

**Table 2:** Total viable count (TVC) in chicken and mutton sample.

Chicken samples	TVC of chicken (log CFU/g)	Mutton samples	TVC of mutton (log CFU/g)
C-1	5.26	M-1	4.48
C-2	5.40	M-2	5.04
C-3	6.46	M-3	4.28
C-4	7.40	M-4	6.26
C-5	8.70	M-5	7.54
C-6	4.83	M-6	4.61
C-7	8.32	M-7	5.18
C-8	4.65	M-8	4.57
C-9	8.34	M-9	6.51
C-10	7.20	M-10	8.28
C-11	7.11	M-11	7.34
C-12	8.61	M-12	8.53
C-13	5.32	M-13	4.58
C-14	5.68	M-14	5.57
C-15	7.04	M-15	6.91
C-16	8.81	M-16	5.46
C-17	6.71	M-17	6.04
C-18	6.20	M-18	5.93
C-19	6.96	M-19	6.26
C-20	8.40	M-20	7.04
C-21	7.58	M-21	8.40
C-22	4.61	M-22	4.49
C-23	6.76	M-23	6.48
C-24	8.81	M-24	7.04
C-25	8.65	M-25	7.32
C-26	4.95	M-26	4.53
C-27	4.88	M-27	4.54
C-28	7.04	M-28	6.65
C-29	6.34	M-29	4.54
C-30	9.40	M-30	7.95
C-31	7.89	M-31	6.04
C-32	5.76	M-32	5.32
C-33	9.46	M-33	8.65
C-34	7.79	M-34	7.04
C-35	4.83	M-35	4.54
C-36	6.78	M-36	5.71
C-37	4.67	M-37	4.49
C-38	4.72	M-38	4.52
C-39	8.45	M-39	7.00
C-40	9.46	M-40	8.48

**Table 3:** Sanitation parameters of the meat outlets.

Outlets code no.	Tiled/cemented	Washing slaughter house		Washing of apron		Washing chopping board		Washing of chopping board for different meat	Use of hand sanitizer	Showcased condition	Water supply area	Drainage	Meat sanitation type
		W	IW	W	UW	W	UW						
1	C	√		√		√		No	No	C	PC	Yes	Good
2	T	√		√		√		No	No	C	PC	Yes	Good
3	T	√		√		√		No	No	C	PC	Yes	Good
4	T	√		√		√		No	No	UC	IC	No	Poor
5	C	√			√	√		No	No	C	IC	No	Good
6	T	√		√		√		No	No	C	PC	Yes	Good
7	C	√			√	√		No	No	UC	IC	Yes	Poor
8	T	√			√	√		No	No	C	PC	Yes	Good
9	C	√			√	√		No	No	UC	IC	No	Poor
10	T	√		√			√	No	No	C	PC	No	Poor
11	C		√		√	√		No	No	UC	IC	Yes	Poor
12	C	√		√			√	No	No	C	PC	Yes	Good
13	C	√		√		√		No	No	UC	PC	No	Good
14	T	√		√		√		No	No	C	PC	Yes	Good
15	C	√		√		√		No	No	UC	IC	Yes	Poor
16	T	√		√		√		No	No	UC	PC	Yes	Poor
17	T	√			√	√		No	No	C	PC	Yes	Poor
18	C		√	√			√	No	No	C	IC	No	Good
19	T	√		√		√		No	No	UC	IC	Yes	Poor
20	C		√	√		√		No	No	UC	IC	Yes	Poor
21	C	√			√	√		No	No	UC	IC	No	Poor
22	T	√		√		√		No	No	C	PC	Yes	Good
23	C		√		√	√		No	No	UC	IC	Yes	Poor
24	T	√		√		√		No	No	UC	IC	No	Poor
25	C	√		√			√	No	No	C	PC	Yes	Good
26	T	√		√		√		No	No	C	PC	Yes	Good
27	T	√			√	√		No	No	C	PC	Yes	Good
28	T	√		√			√	No	No	C	PC	No	Poor
29	C	√		√		√		No	No	UC	PC	No	Good
30	C	√		√		√		No	No	UC	IC	Yes	Poor
31	C	√		√		√		No	No	C	PC	Yes	Good
32	C	√			√	√		No	No	C	IC	No	Good
33	C	√			√	√		No	No	UC	IC	Yes	Poor
34	C	√			√	√		No	No	UC	IC	No	Poor
35	C	√		√		√		No	No	UC	PC	No	Good
36	T	√		√			√	No	No	C	PC	No	Poor
37	T	√		√			√	No	No	C	PC	Yes	Good
38	T	√		√		√		No	No	C	PC	Yes	Good
39	T	√		√		√		No	No	UC	IC	No	Poor
40	T	√		√			√	No	No	C	PC	No	Poor

Observation during sample collection. W,  washed; IW,  improper washing; UW,  unwashed; UC,  uncovered; C,  covered; F, flies observed; PC, properly cleaned; IC,  improperly cleaned.

**Table 4:** Chicken samples vs slaughterhouse/outlets sanitary conditions.

S. no.	Isolates	Outlets		Slaughter house		Apron wash		Chopping box wash		Showcased		Water supply area		Drainage		Meat sanitation	
		Cemented (%)	Tiled (%)	Improperly washed (%)	Properly washed (%)	Unwashed (%)	Washed (%)	Unwashed (%)	Washed (%)	Uncovered (%)	Covered (%)	Improper cleaning (%)	Proper cleaning (%)	Improper (%)	Proper (%)	Poor (%)	Good (%)
1	*Pseudomonas* spp.	65	30	25	50	58.3	42.9	37.5	50	72.2	27.3	70.6	30.4	50	45.8	70	25
2	*E. coli*	85	40	100	58.3	75	57.1	75	59.4	77.8	50	94.1	39.1	75	54.2	85	40
3	*Staphylococcus aureus*	50	55	25	55.6	41.7	57.1	50	53.1	77.8	31.8	58.8	47.8	68.8	41.7	65	40
4	*Staphylococcus epidermidis*	30	50	25	41.7	41.7	39.3	50	37.5	22.2	54.5	17.6	56.5	37.5	41.7	40	40
5	*Salmonella* spp.	65	20	25	44.4	41.7	42.9	62.5	37.5	55.6	31.8	52.9	34.8	56.3	33.3	50	35
6	*Vibrio* spp.	10	0	50	0	8.3	3.6	12.5	3.1	5.6	4.5	11.8	0	6.3	4.2	5	5
7	*Mucor*	40	0	25	19.4	25	17.9	0	25	33.3	9.1	35.3	8.7	18.8	20.8	30	10
8	*Penicillium* spp.	5	45	25	25	8.3	32.1	25	25	16.7	31.8	23.5	26.1	31.3	20.8	25	25
9	*Aspergillus* spp.	10	0	0	5.6	16.7	0	0	6.3	0	9.1	11.8	0	12.5	0	0	10
10	*Sporotrichum* spp.	15	0	0	8.3	25	0	0	9.4	16.7	0	17.6	0	6.3	8.3	15	0
11	*Alternaria* spp.	0	10	0	5.6	16.7	0	0	6.3	0	9.1	0	8.7	0	8.3	0	10
12	*Candida* spp.	0	0	0	0	0	0	0	0	0	0	0	0	0	0	0	0
13	*Trichosporon* spp.	20	15	75	11.1	25	14.3	12.5	18.8	16.7	18.2	23.5	13.0	12.5	20.8	15	20

**Table 5:** Mutton samples vs slaughterhouse/outlets sanitary conditions.

S. no.	Isolates	Outlets		Slaughter house		Apron Wash		Chopping box wash		Showcased		Water supply area		Drainage		Meat sanitation	
		Cemented (%)	Tiled (%)	Improperly washed (%)	Properly washed (%)	Unwashed (%)	Washed (%)	Unwashed (%)	Washed (%)	Uncovered (%)	Covered (%)	Improper cleaning (%)	Proper cleaning (%)	Improper (%)	Proper (%)	Poor (%)	Good (%)
1	*Pseudomonas* spp.	65	35	50	50	58.3	46.4	62.5	46.9	66.7	36.4	76.5	30.4	56.3	45.8	75	25
2	*E. coli*	70	50	75	58.3	66.7	57.1	75	56.3	66.7	54.5	76.5	47.8	68.8	54.2	85	35
3	*Staphylococcus aureus*	40	50	25	47.2	66.7	35.7	50	43.8	55.6	36.4	58.8	34.8	43.8	45.8	60	30
4	*Staphylococcus epidermidis*	45	25	50	33.3	25	39.3	25	37.5	38.9	31.8	23.5	43.5	37.5	33.3	30	40
5	*Salmonella* spp.	50	30	75	36.1	75	25	62.5	34.4	50	31.8	70.6	17.4	75	16.7	65	15
6	*Vibrio* spp.	0	0	0	0	0	0	0	0	0	0	0	0	0	0	0	0
7	*Mucor*	10	15	0	13.9	0	17.9	0	15.6	27.8	0	29.4	0	18.8	8.3	25	0
8	*Penicillium* spp.	10	25	25	16.7	8.3	21.4	12.5	18.8	11.1	22.7	17.6	17.4	6.3	25	10	25
9	*Aspergillus* spp.	15	35	25	25	16.7	28.6	0	31.3	16.7	31.8	17.6	30.4	0	41.7	15	35
10	*Sporotrichum* spp.	10	10	25	8.3	0	14.3	25	6.3	11.1	9.1	11.8	8.7	12.5	8.3	20	0
11	*Alternaria* spp.	10	10	0	11.1	25	3.6	12.5	9.4	0	18.2	11.8	8.7	18.8	4.2	10	10
12	*Candida* spp.	15	0	50	2.8	25	0	0	9.4	16.7	0	17.6	0	6.3	8.3	15	0
13	*Trichosporon* spp.	15	5	0	11.1	25	3.6	12.5	9.4	16.7	4.5	17.6	4.3	12.5	8.3	20	0

**Table 6:** Both chicken and mutton samples vs slaughterhouse/outlets sanitary conditions.

S. no.	Isolates	Outlets		Slaughter house		Apron wash		Chopping box wash		Showcased		Water supply area		Drainage		Meat sanitation	
		Cemented (%)	Tiled (%)	Improperly washed (%)	Properly washed (%)	Unwashed (%)	Washed (%)	Unwashed (%)	Washed (%)	Uncovered (%)	Covered (%)	Improper cleaning (%)	Proper cleaning (%)	Improper (%)	Proper (%)	Poor (%)	Good (%)
1	*Pseudomonas* spp.	65	32.5	37.5	50	58.3	44.6	50	48.4	69.4	31.8	73.5	30.4	53.1	45.8	72.5	25
2	*E. coli*	77.5	45	87.5	58.3	70.8	57.1	75	57.8	72.2	52.3	85.3	43.5	71.9	54.2	85	37.5
3	*Staphylococcus aureus*	45	52.5	25	51.4	54.2	46.4	50	48.4	66.7	34.1	58.8	41.3	56.3	43.8	62.5	35
4	*Staphylococcus epidermidis*	37.5	37.5	37.5	37.5	33.3	39.3	37.5	37.5	30.6	43.2	20.6	50	37.5	37.5	35	40
5	*Salmonella* spp.	57.5	25	50	40.3	58.3	33.9	62.5	35.9	52.8	31.8	61.8	26.1	65.6	25	57.5	25
6	*Vibrio* spp.	5	0	25	0	4.2	1.8	6.3	1.6	2.8	2.3	5.9	0	3.1	2.1	2.5	2.5
7	*Mucor*	25	23.1	12.5	16.7	12.5	17.9	0	20.3	30.6	4.5	32.4	4.3	18.8	14.6	27.5	5
8	*Penicillium* spp.	7.5	35	25	20.8	8.3	26.8	18.8	21.9	13.9	27.3	20.6	21.7	18.8	22.9	17.5	25
9	*Aspergillus* spp.	12.5	17.5	12.5	15.3	16.7	14.3	0	18.8	8.3	20.5	14.7	15.2	6.3	20.8	7.5	22.5
10	*Sporotrichum* spp.	12.5	5	12.5	8.3	12.5	7.1	12.5	7.8	13.9	4.5	14.7	4.3	9.4	8.3	17.5	0
11	*Alternaria* spp.	5	10	0	8.3	20.8	1.8	6.3	7.8	0	13.6	5.9	8.7	9.4	6.3	5	10
12	*Candida* spp.	7.5	0	25	1.4	12.5	0	0	4.7	8.3	0	8.8	0	3.1	4.2	7.5	0
13	*Trichosporon* spp.	17.5	10	37.5	11.1	25	8.9	12.5	14.1	16.7	11.4	20.6	8.7	12.5	14.6	17.5	10

**Table 7:** The chi-square test of different microbial isolates of chicken against several sanitation parameters.

S. No.	Isolates	Outlets tiled/cemented		Slaughterhouse wash		Apron wash		Chop box Wash		Showcase		Water supply area		Drainage		Meat sanitation	
		*χ* ^2^	*p*	*χ* ^2^	*p*	*χ* ^2^	*p*	*χ* ^2^	*p*	*χ* ^2^	*p*	*χ* ^2^	*p*	*χ* ^2^	*p*	*χ* ^2^	*p*
1	*Pseudomonas* spp.	4.912	0.027^*∗*^		0.607^b^	0.807	0.369		0.698^b^	8.021	0.005^*∗*^	6.320	0.012^*∗*^	0.067	0.796	8.12	0.004^*∗*^
2	*E. coli*	8.64	0.003^*∗*^		0.278^b^		0.477		0.686	3.259	0.071	12.61	≤0.001^*∗*^	1.778	0.182	8.64	0.003^*∗*^
3	*Staphylococcus aureus*	0.1	0.752		0.331^b^	0.807	0.369		1^b^	8.386	0.004^*∗*^	0.474	0.491	2.824	0.093	2.506	0.113
4	*Staphylococcus epidermidis*	1.667	0.197		0.638^b^		1^a^		0.69^b^	4.310	0.038^*∗*^	6.155	0.013^*∗*^	0.069	0.792	≤0.001	1
5	*Salmonella* spp.	8.286	0.004^*∗*^		0.624^b^	0.005	0.944		0.25^b^	2.283	0.131	1.319	0.251	2.063	0.151	0.921	0.337
6	*Vibrio* spp.		0.487^b^		0.008^c^		0.515^b^		0.364^b^		1^b^		0.174^b^		1^b^		1^b^
7	*Mucor*		0.003^b^		1^b^		0.677		0.173		0.110		0.053^b^		1^b^		0.235^b^
8	*Penicillium* spp.	8.533	0.008^*∗*^		1^b^		0.231		1^a^		0.464		1^a^		0.556	0.0	1
9	*Aspergillus* spp.		0.487^b^		1^c^		0.085^b^		1^b^		0.492^b^		0.174^b^		0.154^b^		0.487^b^
10	*Sporotrichum* spp.		0.231^b^		1^c^		0.022^b^		1^b^		0.083^b^		0.069^b^		1^b^		0.231^b^
11	*Alternaria* spp.		0.487^b^		1^c^		0.085^b^		1^b^		0.492^b^		0.499^b^		0.508^b^		0.487^b^
12	*Candida* spp.	—	—	—	—	—	—	—	—	—	—	—	—	—	—	—	—
13	*Trichosporon* spp.		1^b^		0.013^b^		0.410^b^		1^a^		1^b^		0.432^b^		0.681^b^		1^b^

*χ* ^2^ value denotes the Pearson chi-square value with degree of freedom (df) = 1. ^a^25%, ^b^50%, and ^c^75% of cells have expected count less than 5, respectively. ^∗^Tested by Fisher's exact test with the level of significance at *p* ≤ 0.05.

**Table 8:** The chi-square test of different microbial isolates of mutton against several sanitation parameters.

S. No.	Isolates	Outlets tiled/cemented		Slaughterhouse wash		Apron wash		Chop box wash		Showcase		Water supply area		Drainage		Meat sanitation	
		*χ* ^2^	*p*	*χ* ^2^	*p*	*χ* ^2^	*p*	*χ* ^2^	*p*	*χ* ^2^	*p*	*χ* ^2^	*p*	*χ* ^2^	*p*	*χ* ^2^	*p*
1	*Pseudomonas* spp.	3.6	0.058		1^b^	0.476	0.490		0.695^b^	3.636	0.057	8.286	0.004^*∗*^	0.417	0.519	10.0	0.002^*∗*^
2	*E. coli*	1.667	0.197		0.638^b^		0.729		0.439^b^	0.606	0.436	3.342	0.068	0.851	0.356	10.417	0.001^*∗*^
3	*Staphylococcus aureus*	0.404	0.525		0.613^b^	3.252	0.071		1^b^	1.473	0.339	2.283	0.131	0.017	0.897	3.636	0.057
4	*Staphylococcus epidermidis*	1.758	0.185		0.602^b^		0.484^a^		0.689^a^	0.218	0.641	1.710	0.191	0.073	0.787	0.44	0.507
5	*Salmonella* spp.	1.667	0.197		0.283^b^		0.005^a^		0.229^b^	1.364	0.243	11.526	0.001^*∗*^	13.611	≤0.001^*∗*^	10.417	0.001^*∗*^
6	*Vibrio* spp.	—	—	—	—	—	—		—	—	—	—	—	—	—	—	—
7	*Mucor*		1^b^		1^c^		0.298^b^		0.563^b^		0.013^b^		0.009^b^		0.373^b^		0.047^b^
8	*Penicillium* spp.		0.407^b^		0.552^b^		0.652^b^		1^a^		0.427^b^		1^b^		0.210^b^		0.407^b^
9	*Aspergillus* spp.	2.133	0.144		1^b^		0.693		0.165^a^		0.464^a^		0.471^a^		0.003^a^	2.133	0.144
10	*Sporotrichum* spp.		1^b^		0.355^c^		0.297^b^		0.172^b^		1^b^		1^b^		1^b^		0.106^b^
11	*Alternaria* spp.		1^b^		1^c^		0.073^b^		1^b^		0.114^b^		1^b^		0.283^b^		1^b^
12	*Candida* spp.		0.231^b^		0.022^c^		0.022^b^		1^b^		0.083^b^		0.069^b^		1^b^		0.231^b^
13	*Trichosporon* spp.		0.605^b^		1^c^		0.073^b^		1^b^		0.310^b^		0.294^b^		1^b^		0.106^b^

*χ* ^2^ value denotes the Pearson chi-square value with degree of freedom (*p*)=1. ^a^25%, ^b^50%, and ^c^75% of cells have expected count less than 5, respectively. ^∗^Tested by Fisher's exact test with the level of significance at *p* ≤ 0.05.

**Table 9:** The chi-square test of different microbial isolates of chicken and mutton meat (in combination) against several sanitation parameters.

S. No.	Isolates	Outlets type		Slaughterhouse wash		Apron wash		Chop box wash		Showcase		Water supply area		Drainage		Meat sanitation	
		*χ* ^2^	*p*	*χ* ^2^	*p*	*χ* ^2^	*p*	*χ* ^2^	*p*	*χ* ^2^	*p*	*χ* ^2^	*p*	*χ* ^2^	*p*	*χ* ^2^	*p*
1	*Pseudomonas*	8.455	0.004^*∗*^	0.45	0.713^b^	1.26	0.262	0.013	0.911	11.22	0.001^*∗*^	14.532	≤0.001^*∗*^	0.409	0.523	18.061	.000^*∗*^
2	*E. coli*	8.901	0.003^*∗*^		0.140^b^	1.327	0.249	1.593	0.207	3.32	0.068	14.403	≤0.001^*∗*^	2.537	0.111	19.013	.000^*∗*^
3	*Staphylococcus aureus*	0.450	0.502		0.265^b^	0.403	0.526	0.013	0.911	8.410	0.004^*∗*^	2.402	0.121	1.201	0.273	6.054	.014^*∗*^
4	*Staphylococcus epidermidis*	0.0	1		1^a^	0.254	0.614	≤0.001	1	1.347	0.246	7.216	0.007^*∗*^	≤0.001	1	0.213	0.644
5	*Salmonella*	8.717	0.003^*∗*^		0.711^b^	4.129	0.042^*∗*^	3.727	0.054	3.589	0.058	10.269	0.001^*∗*^	13.075	.000^*∗*^	8.717	.003^*∗*^
6	*Vibrio* spp.		0.494^b^		0.009^b^		0.513^b^		0.362^b^		1^b^		0.178^b^		1^b^		1^b^
7	*Mucor*	4.501	0.034^*∗*^		1^a^		0.745		0.061	9.843	0.002^*∗*^	11.266	0.001^*∗*^	0.245	0.621	7.44	.006^*∗*^
8	*Penicillium*	9.038	0.003^*∗*^		0.676^a^	3.418	0.064		1^a^	2.119	0.145	0.015	0.901	0.199	0.655	0.672	0.412
9	*Aspergillus*	0.392	0.531		1^a^		0.745^a^		0.111^a^	2.282	0.131	0.004	0.949		0.11^a^	3.529	0.060
10	*Sporotrichum*		0.432^b^		0.536^a^		0.423^b^		0.622^a^		0.234^b^		0.129^b^		1^b^		0.012^b^
11	*Alternaria*		0.675^b^		1^a^		0.008^b^		1^b^		0.03^b^		1^b^		0.679^b^		0.675^b^
12	*Candida* spp.		0.241^b^		0.025		0.025^b^		1^b^		0.087^b^		0.073^b^		1^b^		0.241^b^
13	*Trichosporon*	0.949	0.330		0.075^a^		0.078^a^		1^a^		0.531^a^		0.189^a^		1^a^	0.949	0.33

*χ* ^2^ value denotes the Pearson chi-square value with degree of freedom (*p*)=1. ^a^25%, ^b^50%, and ^c^75% of cells have expected count less than 5, respectively. ^∗^Tested by Fisher's exact test with the level of significance at *p* ≤ 0.05.

**Table 10:** Independent sample *T*-test of mean of different types of isolates in meat against several sanitation parameters and meat type.

Parameters/Factors	Conditions	*N*	Mean	S.D.	S.E.	*T* score	df	*p*	CI lower	CI upper
Outlets	Tiled	40	2.78	1.230	0.194	−3.160	78	0.002^*∗*^	−1.589	−0.361
	Cemented	40	3.75	1.515	0.240					

Slaughter wash	Wash	72	3.19	3.19	0.175	−1.259	78	0.212	−1.757	0.396
	Improper Wash	8	3.88	3.88	0.350					

Apron wash	Wash	56	3.00	1.348	0.180	−2.547	78	0.013^*∗*^	−1.559	−0.191
	Unwashed	24	3.88	1.541	0.315					

Chopping box wash	Wash	64	3.25	1.469	0.184	−0.153	78	0.879	−0.878	0.753
	Unwashed	16	3.31	1.448	0.362					

Showcase	Covered	44	2.77	1.097	0.165	−3.564	78	0.001^*∗*^	−1.696	−0.480
	Uncovered	36	3.86	1.624	0.271					

Water supply	Properly cleaned	46	2.54	1.130	0.167	−6.256	78	≤0.001^*∗*^	−2.230	−1.153
	Improperly cleaned	34	4.24	1.281	0.220					

Drainage	Proper	48	3.00	1.337	0.193	−2.013	78	0.048^*∗*^	−1.305	−0.007
	Improper	32	3.66	1.558	0.275					

Meat sanitation	Good	40	2.38	0.868	0.137	−6.859	78	≤0.001^*∗*^	−2.290	−1.260
	Poor	40	4.15	1.388	0.219					

Meat type	Chicken	40	3.30	1.265	0.2	0.229	78	0.819	−0.577	0.727
	Mutton	40	3.23	1.641	0.259					

df, degree of Freedom. ^*∗*^The level of significance at *p* ≤ 0.05.

**Table 11:** Independent sample *T*-test of mean of TVC in meat against several sanitation parameters and meat type.

Parameters/factors	Conditions	*N*	Mean	S.D.	S.E.	*T* score	df	*p*	CI lower	CI upper
Outlets	Tiled	40	6.057	1.477	0.233	−2.736	78	0.008^*∗*^	−1.547	−0.244
	Cemented	40	6.952	1.449	0.229					

Slaughter wash	Wash	72	6.460	1.581	0.186	−1.362	78	0.193	−1.151	0.254
	Improper wash	8	6.909	0.768	0.272					

Apron wash	Wash	56	6.37	1.542	0.206	−1.216	78	0.228	−1.188	0.287
	Unwashed	24	6.82	1.459	0.297					

Chopping box wash	Wash	64	6.35	1.504	0.188	−1.853	78	0.068	−1.611	0.058
	Unwashed	16	7.126	1.478	0.37					

Showcase	Covered	44	6.048	1.5	0.226	−3.131	78	0.002^*∗*^	−1.662	−0.37
	Uncovered	36	7.064	1.373	0.229					

Water supply	Properly cleaned	46	5.892	1.509	0.222	−4.934	78	≤0.001^*∗*^	−2.021	−0.859
	Improperly cleaned	34	7.333	1.102	0.189					

Drainage	Proper	48	6.262	1.589	0.229	1.773	73.1	0.72	−0.055	1.27
	Improper	32	6.87	1.362	0.24					

Meat sanitation	Good	40	5.6	1.35	0.213	−6.62	78	≤0.001^*∗*^	−2.361	−1.269
	Poor	40	7.412	1.088	0.172					

Meat type	Chicken	40	6.91	1.562	0.247	2.43	78	0.017^*∗*^	0.145	1.46
	Mutton	40	6.103	1.388	0.219					

df, degree of Freedom. ^*∗*^The level of significance at *p* ≤ 0.05.

## Data Availability

All the related data of this study have been included in this manuscript in the tabulated form. These data may help other researchers to replicate or analyze the result of this study.

## Additional Points

Due to limitations of resource, few sanitation parameters, microbial load in outlets' environment and used apparatus, and molecular identifications of isolates could not be performed.
